# A missense MT-ND5 mutation in differentiated Parkinson Disease cytoplasmic hybrid induces ROS-dependent DNA Damage Response amplified by DROSHA

**DOI:** 10.1038/s41598-017-09910-x

**Published:** 2017-08-25

**Authors:** Daniela Pignataro, Sofia Francia, Francesca Zanetta, Giulia Brenna, Stefania Brandini, Anna Olivieri, Antonio Torroni, Giuseppe Biamonti, Alessandra Montecucco

**Affiliations:** 1 Istituto di Genetica Molecolare, Consiglio Nazionale delle Ricerche (CNR), Pavia, 27100 Italy; 2 Dipartimento di Biologia e Biotecnologie “L. Spallanzani”, Università di Pavia, Pavia, 27100 Italy

## Abstract

Genome integrity is continuously threatened by endogenous sources of DNA damage including reactive oxygen species (ROS) produced by cell metabolism. Factors of the RNA interference (RNAi) machinery have been recently involved in the cellular response to DNA damage (DDR) in proliferating cells. To investigate the impact of component of RNAi machinery on DDR activation in terminally differentiated cells, we exploited cytoplasmic hybrid (cybrid) cell lines in which mitochondria of sporadic Parkinson’s disease patients repopulate neuroblastoma SH-SY5Y-Rho(0) cells. Upon differentiation into dopaminergic neuron-like cells, PD63 cybrid showed increased intracellular level of ROS and chronic DDR activation, compared to other cybrids with the same nuclear background. Importantly, DDR activation in these cells can be prevented by ROS scavenging treatment suggesting that ROS production is indeed causative of nuclear DNA damage. Sequence analysis of the mitogenomes identified a rare and heteroplasmic missense mutation affecting a highly conserved residue of the ND5-subunit of respiratory complex I, which accounts for ROS increase. We demonstrated that the assembly of nuclear DDR foci elicited by oxidative stress in these cells relies on DROSHA, providing the first evidence that components of RNAi machinery play a crucial role also in the mounting of ROS-induced DDR in non-replicating neuronal cells.

## Introduction

Genome integrity is continuously undermined by endogenous and exogenous sources of DNA damage^1, 2^. The list of endogenous hazards includes molecular mechanisms that operate on DNA, such as DNA replication and transcription, along with reactive oxygen species (ROS) produced by cell metabolism^3–5^. Cells respond to DNA insults by activating a signalling pathway, the DNA damage response (DDR), which senses the damage and transduces the signal to effector proteins^6^.

The molecular mechanisms that sustain the cell response to DNA damage have been extensively studied in proliferating cells exposed to exogenous sources of DNA damage such as radiations or chemicals. Two main apical protein kinases, ATM (ataxia telangiectasia mutated) and ATR (ATM and Rad3-related kinase), are activated in response to double stranded DNA breaks (DSBs) and stalling of replication forks, respectively. Their activation leads to transient cell cycle arrest and activation of DNA damage repair mechanisms through a multistep signalling cascade mainly regulated by post-translational modifications such as phosphorylation. ATM and ATR phosphorylate a number of mediators such as 53BP1 that build up dynamic molecular complexes called DNA damage response foci where also DNA repair takes place. If the damage is productively repaired, cell proliferation resumes^7^. However depending on the type and amount of DNA damage, cells can fail to recover cell cycle and can either undergo permanent cell cycle arrest, called cellular senescence, or die.

A direct role for RNA in genome surveillance has emerged in the recent years in several different organisms^8, 9^ and DNA damage associated and sequence specific small non coding RNA processed by components of the RNAi machinery have been involved in DDR signalling and DNA repair^10–12^. In particular, small non-coding RNAs complementary to sequences flanking a DSB and whose biogenesis is started by the processing of ribonuclease DROSHA, have been shown to be required for full DDR activation^12–14^.

Contrary to the comprehensive analysis performed in proliferating cells, little is known about the events activating DDR in terminally differentiated cells. Non-replicating cells are hardly replaceable, thus accumulation of DNA damage that leads to cell senescence or apoptosis is detrimental for the organism resulting in loss of tissue functions. This is exemplified in neurodegenerative diseases, in which DNA damage is now considered a hallmark of ageing neurons and neurodegeneration^15, 16^.

Several lines of evidence suggest that sporadic form of Parkinson’s disease (PD) and Alzheimer’s disease (AD) are linked to mitochondrial dysfunction that may arise from mutations or deletions in mitochondrial DNA (mtDNA)^17^. A recent study on the complete spectrum of mtDNA changes, including deletions, copy-number variation and point mutations, in single neurons from the dopaminergic substantia nigra and other brain areas of PD individuals, suggested that dysregulation of mtDNA homeostasis is a key process in the pathogenesis of neuronal loss in Parkinson disease^18^.

The mitochondrial respiratory chain consists of four multi-subunit complexes named I (NADH:ubiquinone oxidoreductase, CI), II (succinate dehydrogenase, CII), III (cytochrome *bc1* complex, CIII), and IV (cytochrome *c* oxidase, CIV) that reside in the inner mitochondrial membrane and play a major role in energy conversion. CI, CIII and CIV establish the proton gradient across the mitochondrial membrane that can be employed by the ATP synthase (complex V) to drive ATP synthesis^19^. Dysfunction of mitochondrial respiratory chain complexes generates reactive oxygen or nitrogen species that may cause persistent DNA damage, neuronal impairment and death leading to neurodegenerative diseases (NDDs), a group of disorders, including AD and PD^18, 20^, characterized by the accumulation of various types of genomic DNA damage^15^.

Cytoplasmic hybrid (cybrid) cell lines have been extensively used to explore the relevance of mitochondrial dysfunction in PD and AD^21^. In particular human neuroblastoma (SH-SY5Y) cells that are made deficient in mtDNA, termed Rho(0), can be repopulated with mitochondria from AD and PD patients to form cybrids^22^. Since cybrids share a common genomic DNA, but different mitogenomes, they provide a model to understand the impact of mitochondrial DNA mutations on neuronal cells. PD cybrid lines described so far exhibit several features of mitochondrial dysfunction such as decreased mitochondrial electron transport chain (mtETC) complex activity, increased number of morphologically abnormal mitochondria and higher production of ROS that cause oxidative stress to the cells^21^.

In this study, to evaluate the response of differentiated cells to endogenous sources of DNA damage caused by mitochondrial dysfunction, we exploited a previously described cybrid cell line, PD63^21^ bearing the mtDNA from a sporadic PD patient. We show that the mitogenome of this cybrid harbours an extremely rare missense mutation in the ND5 (NADH-ubiquinone oxidoreductase chain 5) subunit of the CI complex. Upon differentiation to dopaminergic neuron-like cells, PD63 shows an increased intracellular level of ROS and has chronic DNA damage and DDR activation. We also observed that the assembly of DDR foci elicited by oxidative stress relies on DROSHA endonuclease, providing the first evidence that components of the RNAi machinery play a crucial role in the cellular response to endogenous sources of DNA damage induced by oxidative stress in differentiated neuronal-like cells.

## Results

### Phosphorylation of histone H2AX in differentiated cybrids from PD patients

An emerging theme is that Parkinson’s disease could be linked to persistent genomic DNA damage caused by oxidative stress^23^. To assess the impact of mitochondrial dysfunction on genome integrity we analysed markers of the DDR in cybrids obtained from sporadic PD patients. The analysis was performed after inducing neuronal differentiation in order to avoid possible DNA damage resulting from DNA replication.

**Figure 1:**
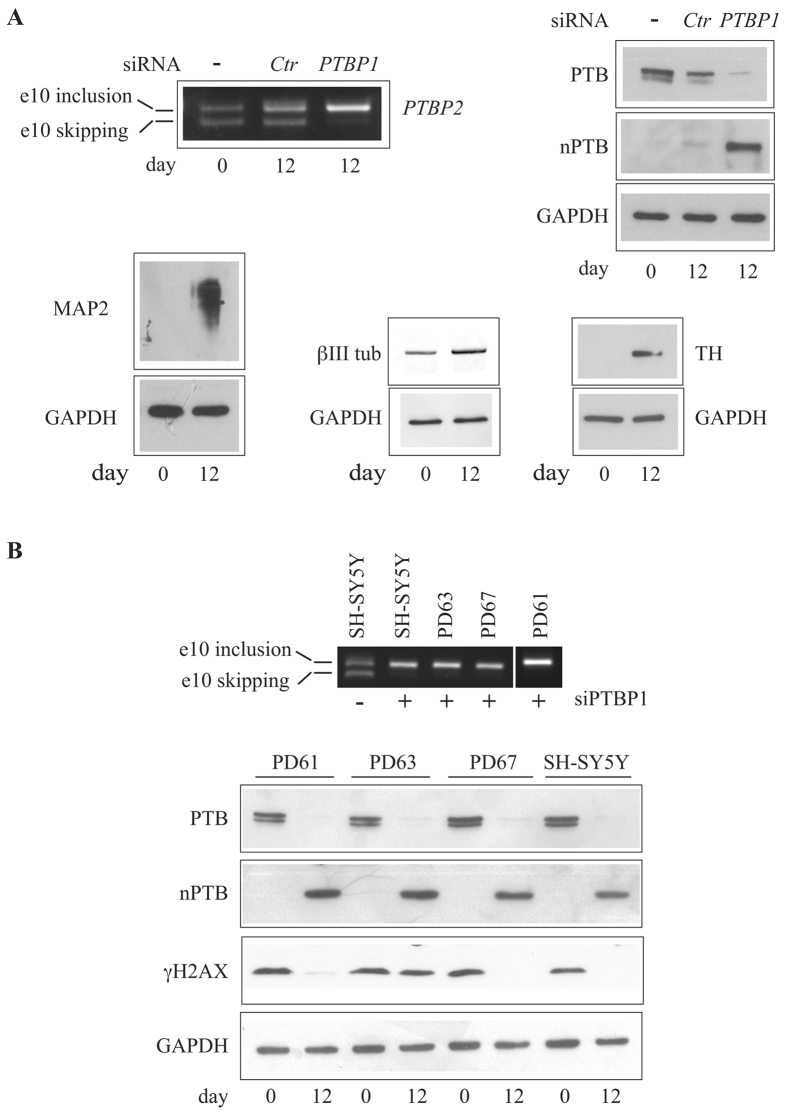
SH-SY5Y cells and cybrids were differentiated to dopaminergic neuron-like cells. (**A**) SH-SY5Y cells were grown in differentiation media as described in Materials and Methods. Upper panels show the alternative splicing profile of *PTBP2* exon 10 before (day 0) and after differentiation (day 12) (left) and the level of PTB and nPTB proteins (right). In the left panel, the upper band shows the full-length *PTBP2* that includes exon 10 while the lower band shows the *PTBP2* transcript produced by exon 10 skipping. At day 1, cells were transfected with control siRNA (Ctr) or with siRNA directed against *PTBP1* mRNA. Lower panels show Western blot analyses of three neuronal differentiation markers, microtubule-associated protein 2 (MAP2), beta-3-tubulin (βIII tub) and tyrosine hydroxylase (TH), on whole SH-SY5Y cell lysates before (day 0) and after (day 12) differentiation. GAPDH was used as loading control. (**B**) PD61, PD63, PD67 cybrids and SH-SY5Y cells were grown in differentiation media. At day 12 total RNA was purified and the alternative splicing profile of *PTBP2* exon 10 was analysed by RT-PCR (upper panel). Lower panel shows the Western blot analyses of PTB, nPTB and phosphorylated histone H2AX (γH2AX) before and after differentiation on the indicated cell lines. GAPDH, loading control.

**Figure 2:**
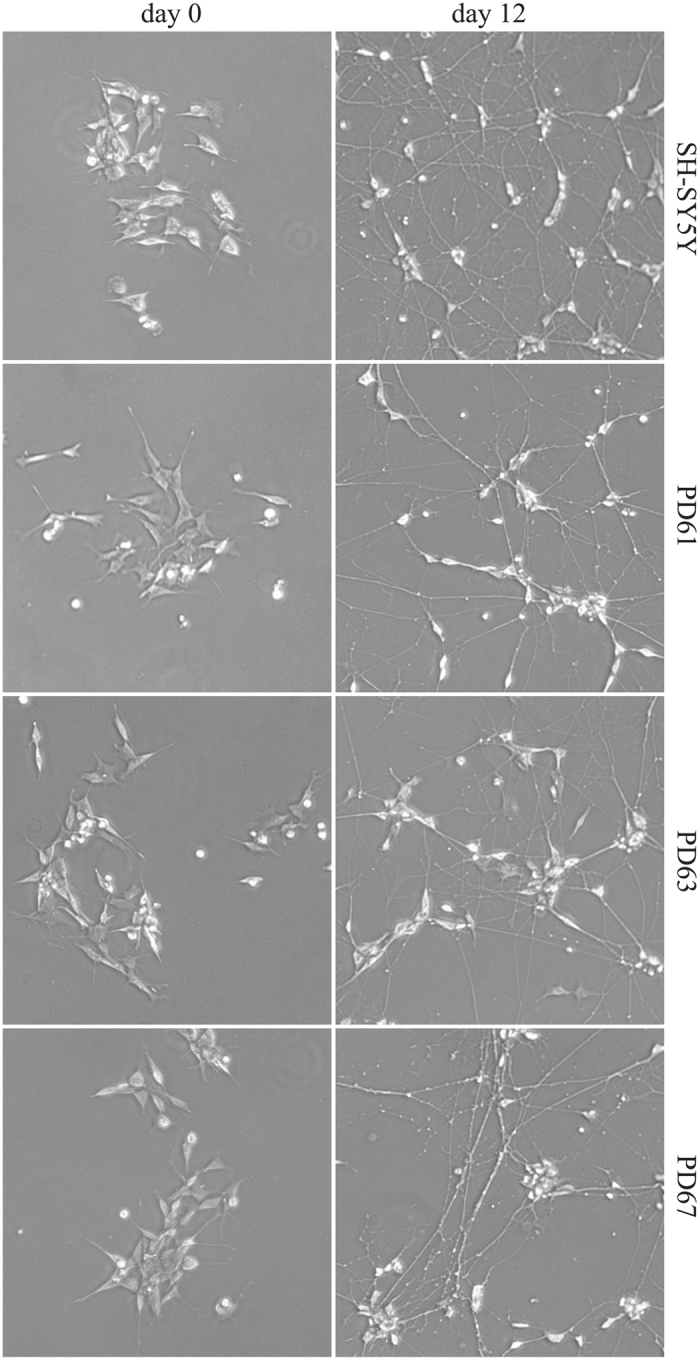
Bright-field microscopy images (10X) of proliferating (day 0) and differentiated (day 12) cells. SH-SY5Y neuroblastoma cells and cybrids (PD61, PD63, PD67) were differentiated to neurone-like cells after siRNA-mediated silencing of *PTBP1*.

Cybrids used in this study (PD61, PD63, PD67) were created by repopulating human SH-SY5Y neuroblastoma cells, previously depleted of endogenous mtDNA [Rho(0) cells], with platelet mtDNAs from three sporadic PD patients^22, 24^. Mitochondrial biogenesis and composition of respirasome in these cybrid cell lines were previously characterized^25^. To obtain neuron-like cells, we applied a differentiation protocol that couples standard treatment with growth factors to siRNA-mediated down-regulation of splicing factor PTBP1. It has been reported that the switch between two splicing regulators, the ubiquitously expressed PTBP1 (also known as poly-pyrimidine binding protein or hnRNP-I) and its neuronal paralog PTBP2 (or nPTB), is crucial to neuronal differentiation^26^. Interestingly, PTBP1 prevents PTBP2 expression by inducing an unproductive splicing event that directs the *PTBP2* mRNA toward degradation by the non-sense mediated RNA decay (NMD) pathway. *PTBP1* down-regulation is sufficient to induce *PTBP2* expression. However, by following the standard differentiation protocol based on treatment with a cocktail of differentiation factors (5 days of retinoic acid (RA) followed by 7 days in the presence of BDNF, neuregulin, NGF, N2 and vitamin D3), we were unable to obtain in differentiated SH-SY5Y cells the expected PTBP1/PTBP2 switch. Therefore, we applied a protocol in which the treatment with differentiation factors was preceded by siRNA-mediated silencing of *PTBP1*. This treatment can induce the expression of the full-length *PTBP2* transcript (exon 10 included) lasting for the entire differentiation period (12 days) (Fig. 1A). Western blot analysis of proliferating and differentiated cell extracts confirmed the occurrence of the PTBP1/PTBP2 switch in SH-SY5Y cells (Fig. 1A). We also verified the expression of neuronal markers such as MAP2 and βIII tubulin that increase in differentiated cells and the expression of tyrosine hydroxylase, which is a marker of dopaminergic neuronal differentiation (Fig. 1A). Based on these findings we applied the same protocol to PD61, PD63 and PD67 cybrids. As shown in Fig. 1B, at the end of the differentiation protocol (day 12), all the cybrids expressed the full-length *PTBP2* transcript and the neuronal specific PTBP2 protein. The morphology of SH-SY5Y and cybrids before and after differentiation is shown in Fig. 2. At day 12 cells show a remarkable increase of their neuronal processes and tend to form clustered somata as expected for neuronal-like cells.

A DDR hallmark is the phosphorylation of histone H2AX on Ser139 (γH2AX) by activated and autophosphorylated p-ATM DDR kinase^27^. In order to investigate H2AX phosphorylation, we performed Western blot analysis of whole cell lysates from proliferating and differentiated neuroblastoma SH-SY5Y cells and cybrids. An increased basal level of histone H2AX phosphorylation is known to occur in tumour cells during S phase due to replicative stress^28^. Indeed, as shown in Fig. 1B, histone H2AX is phosphorylated in proliferating neuroblastoma cells and all the cybrids, possibly due to replication stress. Consistent with this interpretation, γH2AX drastically decreased in differentiated non-proliferating SH-SY5Y. A comparable decrease in γH2AX level was detectable in differentiated PD61 and PD67 cells, while in PD63 an intense γH2AX signal was observed even after neuronal differentiation suggesting persistent accumulation of DNA damage.

### A missense heteroplasmic mutation in MT-ND5 of the PD63 cybrid

All cybrids harbour the SH-SY5Y nuclear genome but differ in their mtDNA. To assess whether the different phenotype of the PD63 cells could be due to the sequence variation of its mtDNA, we PCR-amplified the entire mtDNA of PD63 as well as those of PD61 and PD67, and completely sequenced their mitogenomes following a well-established Sanger protocol^29^. We aligned, assembled, and compared the three sequences using Sequencher 5.0 (Gene Codes Corporation). For each mitogenome, all coding- and control-region mutations are reported in Table 1.

**Table 1:** Map location of the mutations characterizing the mitogenomes of the PD61, PD63 and PD67 cybrids.

MAP Locus^a^	PD61^b^ GenBank: KY498629 Haplogroup: L2e1a	PD63^b^ GenBank: KY498630 Haplogroup: H1a3b	PD67^b^ GenBank: KY498631 Haplogroup: H3b1b1
MT-RNR1	G719A A750G G769A C954T G1018A A1171h G1211A A1438G	A750G A1438G	A750G A1438G
MT-RNR2	T2416C A2706G	G3010A	A2581G
MT-ND1	A3537G C3594T C4086T A4104G		
MT-ND2	A4562G A4769G A5069T (M200I)	A4769G	A4769G G5147A
MT-CO1	T6014C C6617T C7028T C7256T		T6776C A7403G
MT-TD	G7521A		
MT-ATP8	T8383C		
MT-ATP6	A8701G (T59A) A8860G (T112A) G8994A	A8860G (T112A)	A8860G (T112A)
MT-CO3	A9221G A9377G T9540C C9971T		
MT-ND3	T10115C A10398G (T114A)		
MT-ND4	G10873C G11149A G11719A T11935C A11989G		
MT-TH	T12189C		
MT-TS2	G12236A		
MT-ND5	C12705T G13194A G13590A C13650T G13708A (A458T)	G13804h (A490T)	G13813A (V493I)
MT-ND6	C14266T T14299C		
MT-CYB	C14766T (T7S) G15301A A15326G (T194A) T15697C G15734A (A330T)	A15326G (T194A)	A15326G (T194A)
MT-TT	T15889C		
MT-DLOOP	C16111A G16145A C16184T T16189C C16223T C16239T C16278T C16292T C16355T G16390A A16399G C16400T T16519C A73G T146C C150T T152C C182T A183G A263G 309.1 C 315.1 C A479G	A16051G A16162G A16241C C16465T T16519C A73G T195C A263G 315.1 C A522d C523d	C16111T G16129A C16256T T16519C T146C A153G A263G 309.2 C 315.1 C

^a^Map locus nomenclature is according to https://www.mitomap.org/MITOMAP.

^b^Mutations are relative to the revised Cambridge Reference Sequence (rCRS)^61^. Heteroplasmic nucleotide positions are marked by an “h”, deletions by a “d”, while insertions are indicated by “.” followed by number and type of inserted nucleotide(s). All observed mutations in the mtDNA coding region (from np 577 to np 16023) are diagnostic of the corresponding haplogroup except those underlined. For non-synonymous mutations the amino acid change is reported in brackets.

The observed mutational motifs allowed the classification of the three mitogenomes into well-defined haplogroups (see: Phylotreee: http://www.phylotree.org/)^30^. The PD61 mitogenome is a member of the sub-Saharan haplogroup L2e1a, while PD63 and PD67 are characterized by haplogroups H1a3b and H3b1b1, respectively, which are typical of western European populations. For all mitogenomes, almost all coding-region mutations are diagnostic of the corresponding haplogroup, i.e. they are shared by all haplogroup members. The private coding-region mutations observed in PD61 were six (1171, 4086, 6617, 11989, 12236 and 14266) and only one each in PD63 (13804) and PD67 (7403) (Table 1). Of these only the 1171 mutation of PD61 is in the 12rRNA gene, whereas all others are synonymous mutations in protein coding genes, except the only one found in PD63. Indeed, the PD63 mitogenome contains an extremely rare missense mutation at np 13804 (G to A) in the codon 490 of the complex I subunit ND5 (MT-ND5) that causes an amino acid change, from alanine to the hydrophilic threonine (m.13804 G > A, p.Ala490Thr), in the 14th trans-membrane α-helix (http://www.uniprot.org/uniprot/P03915) at the C-terminus of the protein. In PD63 the m.13804 G > A (p.Ala490Thr) mutation was found in a heteroplasmic state, i.e. mitogenomes with and without the mutation were co-present, with a prevalence (about 70%) of mutant mitogenomes. The percentage of mutant mtDNA molecules was better assessed at a value of 66% (data not shown) by Next Generation Sequencing (NGS) with an Illumina MiSeq^®^. So far the m.13804 G > A (p.Ala490Thr) has been reported only once (Mitomap: http://www.mitomap.org/MITOMAP) in a mitogenome belonging to a different haplogroup (HV0) from a subject included in a general population survey^31^. An a posteriori investigation of that finding revealed that the subject with the m.13804 G > A (p.Ala490Thr) mutation is an health 58 years old male from Italy, whose mtDNA (from blood), intriguingly, was also found to be heteroplasmic, but at much lower level (33% with NGS) than that observed in PD63 (Luiselli D. and De Fanti S., personal communication). If we take into account that the most severe mtDNA mutations are only detected in a heteroplasmic state in patients, probably because they are lethal when homoplasmic, and the bio-energetic defect caused by a mtDNA mutation becomes evident only when a certain tissue-specific threshold of mutant mtDNA is reached^32^, our observations fit the scenario that the m.13804 G > A mutation might be the cause of a major bio-energetic defect.

### The PD63 cybrid with the m.13804 G > A (p.Ala490Thr) MT-ND5 mutation activates a ROS-dependent chronic DNA damage response

**Figure 3:**
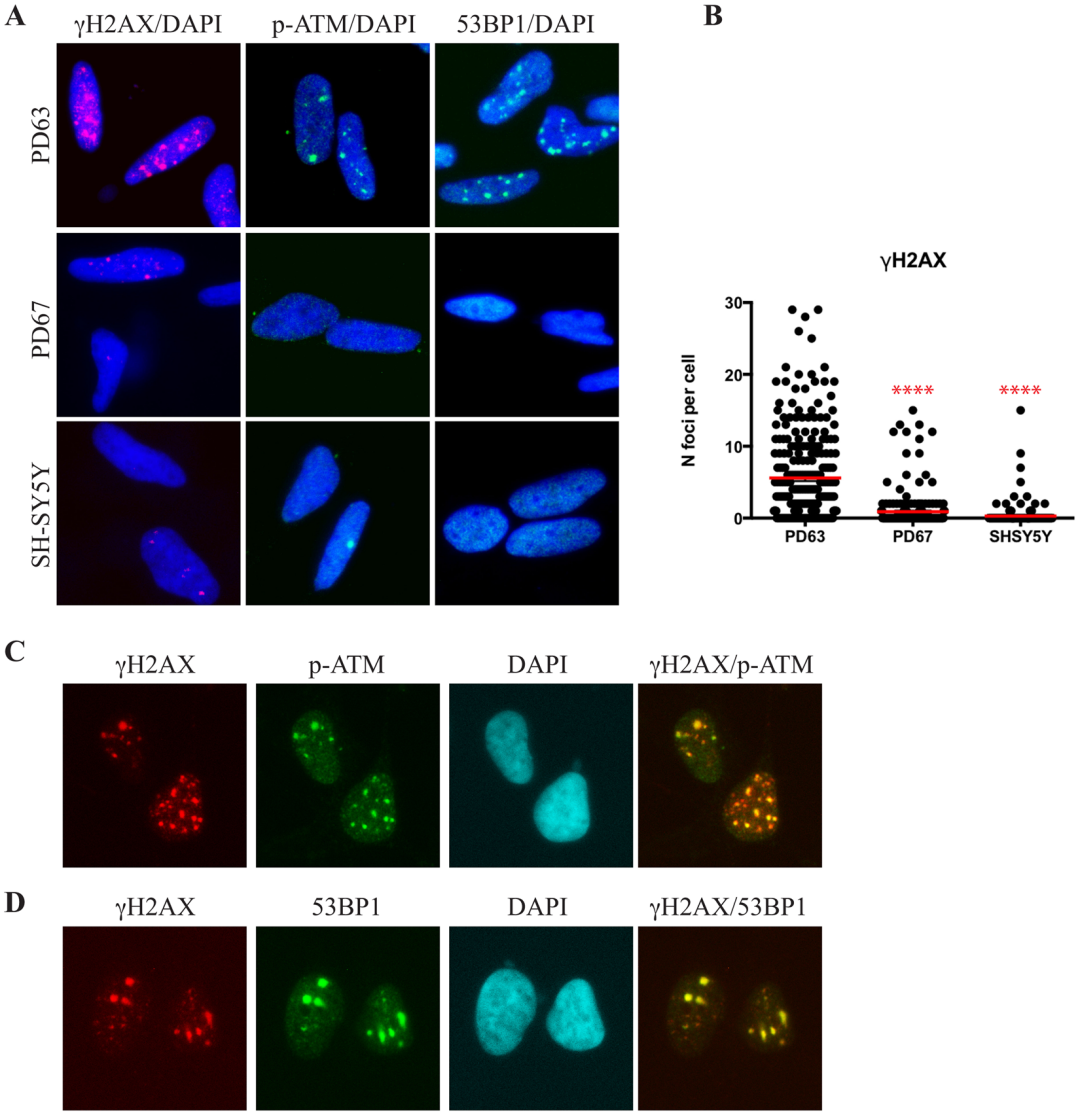
DNA repair foci accumulate in differentiated PD63 cybrids. (**A**) Immunolocalization of γH2AX (red), ATM^pS1981^ (p-ATM) (green) and 53BP1 (green) in PD63, PD67 and SH-SY5Y cells after differentiation. Cells were seeded and differentiated on coverslips and immunostained with specific antibodies. (**B**) The number of foci per nucleus in the three cell lines was quantified with CellProfiler as described in Material and Methods. Red lines indicate the means of γH2AX foci per nucleus. For these analyses more than 200 cells for each condition were analysed; **** = p < 0.0001. (**C**) Immunofluorescence showing the colocalisation of γH2AX (red) and ATM^pS1981^ (p-ATM) (green) in differentiated PD63 cybrid cells. (**D**) Immunofluorescence showing the co-localization of γH2AX (red) and 53BP1 (green) in differentiated PD63 cybrid cells. Nuclei were stained by DAPI.

Phosphorylation of H2AX spreads on the chromatin surrounding the DNA lesion and is a signal for the recruitment of DDR mediator proteins at the site of damage forming cytologically detectable nuclear foci positive for 53BP1 and activated ATM^33^. In order to characterize the DDR in cells with the 13804 G > A (p.Ala490Thr) missense mutation, PD63 cybrid cells were seeded on coverslip-containing Petri dish and induced to differentiate. At day 12 coverslips were immunostained for the DDR markers γH2AX, p-ATM and 53BP1. In parallel, PD67 cybrid cells, that did not show mutations in their mitogenomes, and SH-SY5Y cells were also analysed as experimental control. In agreement with the Western blot analysis in Fig. 1B, discrete foci of γH2AX, p-ATM and 53BP1 were clearly detectable in PD63 cells (Fig. 3A). The quantification showed that the number γH2AX nuclear foci after differentiation, was significantly higher in PD63 compared to PD67 and SH-SY5Y cells (Fig. 3B). Double staining immunofluorescence showed the colocalization of p-ATM kinase and 53BP1 at γH2AX foci in PD63 cells (Fig. 3C,D), demonstrating that the DDR is chronically activated. This finding is a strong indication that chromosomal DNA damage accumulates in non-replicating differentiated PD63 cybrid cells.

**Figure 4:**
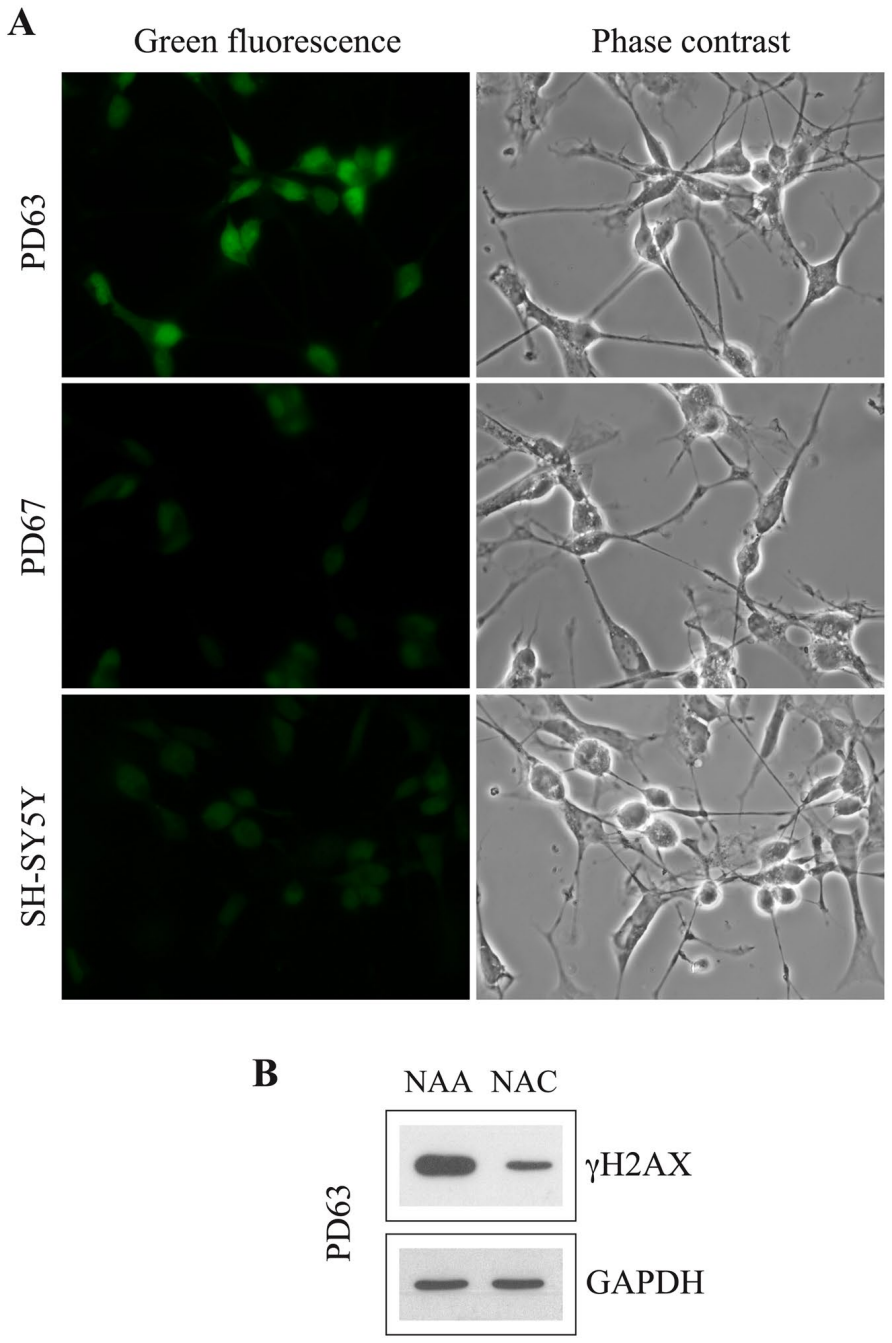
Differentiated PD63 cybrid cells have increased levels of ROS. (**A**) ROS detection (green) with CellROX Green Reagent kit (Invitrogen™). Phase contrast images (60X) of the same fields are also shown. (**B**) Western blot analysis of γH2AX in differentiated PD63 cells grown in the presence of N-acetyl-cysteine (NAC) or N-acetyl-alanine (NAA) as control. GAPDH, loading control.

Since the m.13804 G > A (p.Ala490Thr) mutation causes an amino acid change in the codon 490 of MT-ND5 and the possible impairment of complex I could result in an increased level of cytoplasmic ROS, we investigated the ROS level in PD63 cybrid cells. Intracellular ROS accumulation in the form of superoxide anions can be detected by fluorescence microscopy using the cell ROS probe (CellROX Green reagent) that, upon oxidation, exhibits fluorogenic signal. We observed that PD63 cells indeed accumulated increased level of ROS compared to both PD67 and SH-SY5Y suggesting a correlation between the m.13804 G > A (p.Ala490Thr) mutation and the mitochondrial dysfunction (Fig. 4A). Consistent with a chronic oxidative stress the level of malondialdehyde (MDA), which is the main product resulting from lipid peroxidation, is higher in PD63 than in PD67 and SH-SY5Y cells (see Supplementary Table 1S). As a functional parameter of mitochondrial activity we measured the ATP content in PD63, PD67 and SH-SY5Y cells. While SH-SY5Y and PD67 have approximately the same ATP content (1.8 and 2.4 × 10^−15^ moles of ATP per cell respectively) this value is 3-4 fold lower (0.6 × 10^−15^ moles per cell) in PD63 cells. This finding indicates an impairment of mitochondrial function in cybrid cells with the rare m.13804 G > A mutation.

Persistent oxidative stress can also damage nuclear DNA. We therefore investigated whether increased level of ROS in PD63 cells could cause DNA damage and H2AX phosphorylation. To this aim, we treated cells with *N*-acetyl-cysteine (NAC), a broad-specificity ROS scavenger that acts by replenishing cellular glutathione; as a negative control, we used *N*-acetyl-alanine (NAA), a related but inactive compound. Western blot and immunofluorescence analysis showed that NAC addition to culture medium reduced H2AX phosphorylation in differentiated PD63 cells (Fig. 4B and Supplementary Fig. 1S). Therefore, we conclude that oxidative stress causes H2AX phosphorylation in MT-ND5 mutated PD63 cybrid cells.

### DROSHA contributes to the DNA damage response activated by ROS-mediated DNA damage

**Figure 5:**
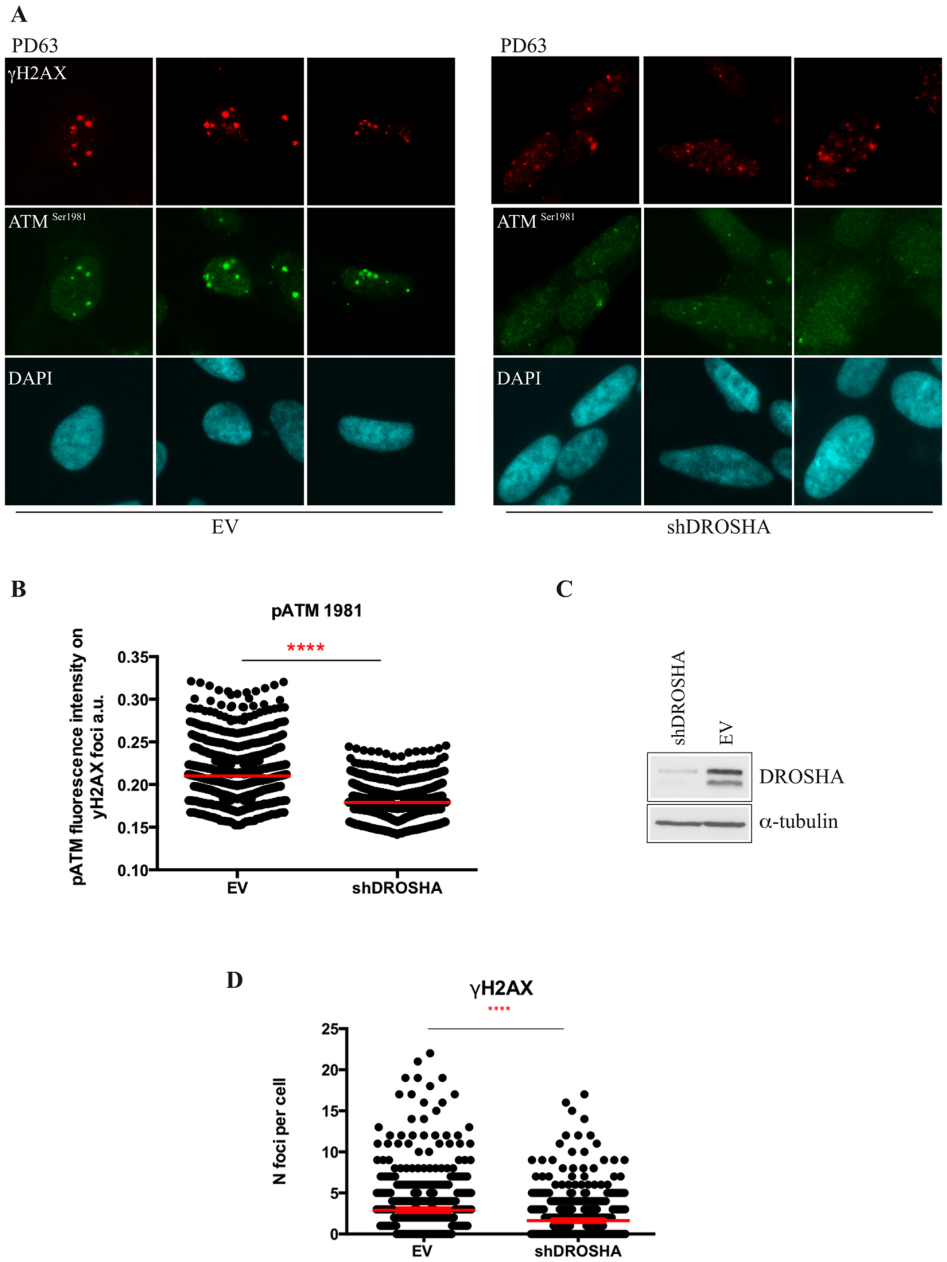
Endoribonuclease DROSHA contributes to the recruitment of p-ATM to γH2AX foci in differentiated PD63 cells. (**A**) Immunolocalisation of γH2AX (red) and ATM^pS1981^ (green) in differentiated PD63 cybrids infected with lentiviruses expressing shRNA targeting DROSHA (shDROSHA) or pLKO empty vector as control (EV). Nuclei were stained by DAPI. (**B**) Quantification of the ATM^pS1981^ (pATM) in γH2AX foci in PD63 infected with lentiviruses expressing the empty vector (EV) or shDROSHA. Red lines indicate the mean of pATM focus intensity in the γH2AX-positive damaged area. (**C**) Western blot analysis of endoribonuclease DROSHA level in PD63 infected with lentiviruses expressing shDROSHA or the empty vector (EV); α-tubulin, loading control. (**D**) The number of γH2AX foci per nucleus in PD63 infected with lentiviruses expressing the empty vector (EV) or shDROSHA was quantified with CellProfiler as described in Methods. Red lines indicate the means of γH2AX foci per nucleus. For these analyses more than 300 cells for each condition were analysed ****=p < 0.0001.

It has been previously demonstrated^13, 14^ that endoribonuclease DROSHA contributes to DDR activation by starting the process that generates small non-coding RNAs at sites of DNA damage, called DDRNAs. These molecules promote the recruitment of DDR factors into cytologically detectable foci and amplify the signal downstream of γH2AX at sites of DNA damage^13^. This mechanism has been mainly characterized in proliferating cells after acute DNA damage induced by ionizing radiation, radiomimetic drugs or nuclease activity and only marginally has been addressed the impact of DROSHA on DNA damage induced by oncogene activation^13, 14^. Certainly, the involvement of DROSHA in DDR signal amplification in post mitotic neuronal cells exposed to oxidative stress has not been tested yet. Therefore, we investigated the contribution of DROSHA to the ROS-induced DDR foci formation in differentiated PD63 cells. To this aim, DROSHA was knocked-down by shRNA in differentiated PD63 cells and the impact on p-ATM recruitment to γH2AX positive foci was tested by immunostaining. PD63 cells were seeded on a coverslip-containing Petri dish and differentiated. 48 h before processing, cells were then infected with shDROSHA-expressing lentiviral particles or empty vector carrying viruses (EV), as control. At day 12, coverslips were stained for γH2AX and p-ATM while cells grown on the Petri dishes were collected and immunoblotted to verify DROSHA expression levels in control and knock down samples (Fig. 5C). Remarkably, DROSHA inactivation significantly impacts on p-ATM recruitment to γH2AX foci in differentiated PD63 cells (Fig. 5A,B). It is known that activated ATM is required for the spreading of H2AX phosphorylation^6^ and the amplification of DDR signal. Thus, an impairment in p-ATM recruitment is expected to reduce the number of γH2AX foci detectable by immunofluorescence. In agreement with this, DROSHA down-regulation in terminally differentiated cells, by impairing the recruitment of p-ATM, also significantly reduced the number of γH2AX foci compared to cells transfected with the empty vector (EV) (Fig. 5D). Importantly, total H2AX transcript and protein level remained unaffected upon DROSHA knock down (Supplementary Fig. 2S), ruling out an indirect effect on H2AX gene transcript due to loss of mature microRNAs in DROSHA inactivated cells or other microRNA independent functions of DROSHA. DROSHA acts upstream of DICER during both DDRNA and microRNA biogenesis^13, 14^. Indeed, a reduction of DDR foci, although to a lesser extent, was also obtained upon DICER down regulation (Supplementary Fig. 3S). Altogether these observations suggest that DROSHA is required for DDR activation in response to chronic oxidative stress in terminally differentiated cells and points to this factor as a novel player in the stress response associated with mitochondrial dysfunctions in PD.

## Discussion

In this study we exploited previously isolated and characterized PD cybrids^21, 22^ to investigate the effect of mitochondrial dysfunction on DDR activation, which in turn could drive neuronal aging and neurodegeneration. To study the impact of mitochondrial dysfunction on DNA damage, we performed our analysis in parental SH-SY5Y cells and PD cybrids upon differentiation into non-dividing dopaminergic neuronal cells, which are the main target of neurodegeneration in PD. In previous studies PD cybrids were differentiated in a medium containing staurosporin^21^, a broad spectrum protein kinase inhibitor that affects cell cycle checkpoint interfering with the phosphorylation of DNA repair factors^34, 35^. Therefore, to investigate the DDR in differentiated cybrids, we applied a different protocol previously used for SH-SY5Y cells differentiation^36^. We observed that standard *in vitro* differentiation of SH-SY5Y cells driven by a cocktail of growth factors was unable to achieve the physiological switch between splicing factor PTB (encoded by *PTBP1* gene) and its neuronal counterpart (nPTB) encoded by *PTBP2* gene. Therefore, we applied a modified protocol in which siRNA–mediated *PTBP1* down-regulation was performed before treatment with the differentiation media. Remarkably, it has been shown that siRNA–mediated *PTBP1* down-regulation is sufficient to promote nPTB expression and to allow trans-differentiation of fibroblasts into neuron-like cells^26^. Consistent with this observation we could trigger the switch to the neuron specific nPTB splicing factor both in SH-SY5Y and in all cybrid cell lines used in this study (Fig. 1).

In agreement with the idea that DNA replication is a main cause of DNA damage and, hence, of DDR activation in proliferating tumour cells^37^, the level of γH2AX in parental SH-SY5Y cells and in two cybrids (PD61 and PD67) drastically decreased after post-mitotic differentiation. In contrast, the γH2AX level remained high in differentiated PD63 suggesting a chronic DDR activation independent from S-phase. Moreover, foci containing γH2AX, pATM and 53BP1 were clearly detectable in these cells indicating the assembly of functional DDR complexes.

Several lines of evidence collected here suggest that chronic DDR activation in PD63 cells may be linked to oxidative stress caused by pathological mitochondria. Indeed, it has been previously shown that the mtETC complex is not correctly assembled in PD63 cells due to an impairment of mitochondrial complexes I and IV^21^. This may be due to a mutation in one of the several mitochondrially-encoded subunits of these complexes.

**Figure 6:**
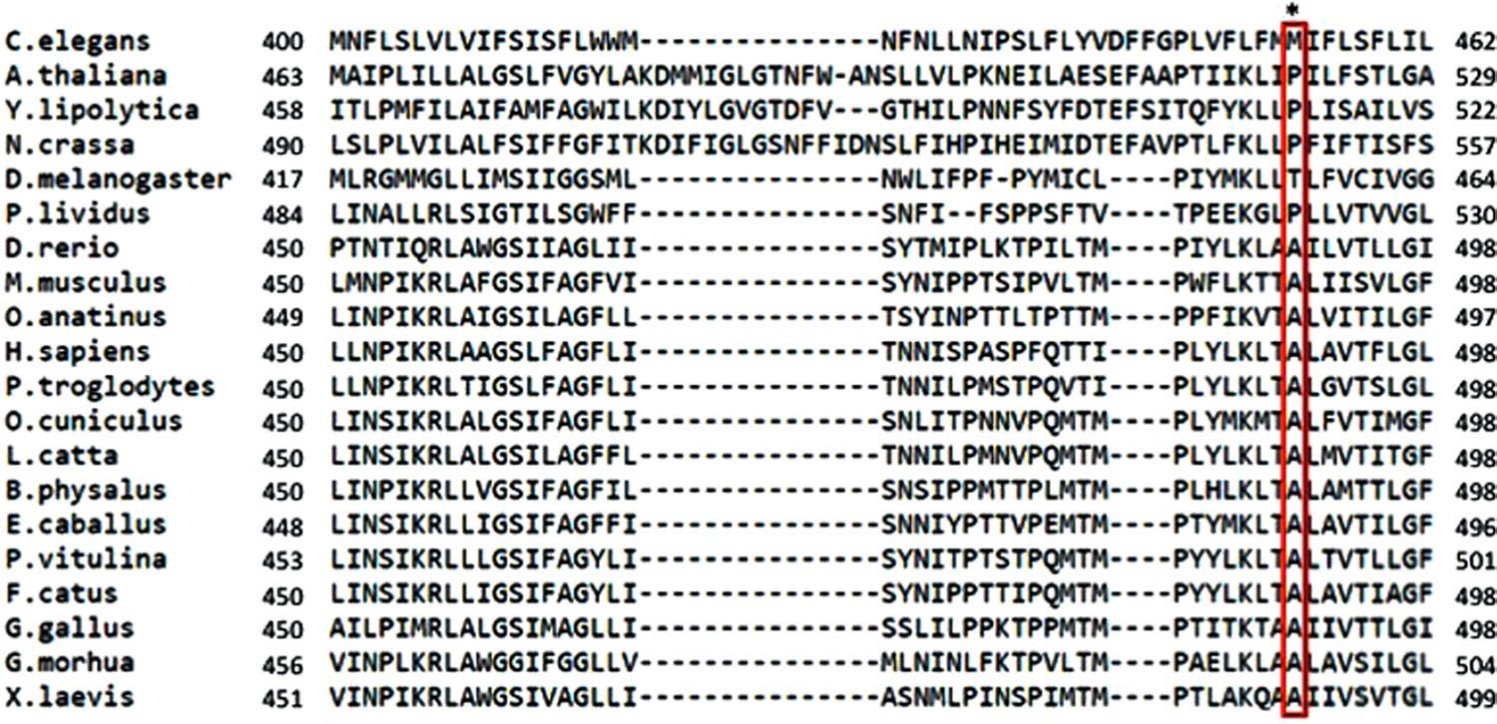
The global alignment of ND5 protein sequences from a wide range of eukaryotes. The alanine at codon 490 in humans (marked by an asterisk) is a shared and extremely conserved feature among all vertebrates.

By complete sequencing of the PD63 mitogenome we identified an extremely rare missense mutation (m.13804 G > A, p.Ala490Thr) which causes an amino acid change, from alanine to the hydrophilic threonine, at codon 490 of the complex I subunit ND5 (MT-ND5), within the 14^th^ trans-membrane α-helix at the C-terminus of the protein (http://www.uniprot.org/uniprot/P03915). The alanine at codon 490 of the ND5 subunit is a shared and extremely conserved feature among all vertebrates (Fig. 6), whose appearance occurred in the Late Cambrian, i.e. about 500 Myr ago, according to both fossil records and molecular data^38^.

An additional feature of the m.13804 G > A (p.Ala490Thr) mutation in PD63 is its heteroplasmic state, with a load of 66% mutant mitogenomes. Intriguingly, our *a posteriori* investigation revealed a heteroplasmic state also in the only other instance in which this mutation was reported. It was in a health 58 years old male, but at a much lower mutational load (33% with NGS). Note that a threshold effect and a detection exclusively in heteroplasmy are both well-known features of severe disease-causing mtDNA mutations^32^.

In contrast, no mutations were detected either in MT-ND5 or in the other components of the mtETC complex in PD61 and PD67 mitogenomes. A mutation in MT-ND5 (m.12955 A > G) located in the third hydrophilic extra-membrane loop, which impairs complex I assembly and reduces complex IV stability, has been recently described in an OXPHOS deficient patient^39^. Although the m.12955 A > G mutation is causative of the disease, the pathological condition and the elevation of ROS are largely dependent on the mutant load. Indeed, the patient showed a higher fraction of mutated mtDNAs than his healthy mother. At the molecular level, studies with cybrids proved that mitochondrial complex I and IV activities are reduced in cells with 98% of m.12955 A > G mtDNAs, but are apparently normal in cells bearing up to 65% of the mutant mtDNAs. This impairment of complexes I and IV results in a higher level of ROS and cytotoxicity.

In line with these data, we found that differentiated PD63 cells, containing 66% of mutated mitogenomes, have an increased level of ROS compared to SH-SY5Y and PD67 cells (Fig. 4).

Although the original cells of the PD patient, from whom the PD63 mitogenome has been obtained, are no longer available thus precluding further studies, we are tempted to propose that the 66% load of m. 13804 G > A (p.Ala490Thr) mutation in MT-ND5 that we observed in PD63 cells, leads to neurodegenerative disorders by increasing the ROS level and activating the DDR. Contrary to mutations in other subunits of respiratory complex I, such as ND1, ND2, ND4, and ND6, which have been proposed to hamper complex I assembly, defects in ND5 have been reported to deeply impact enzyme activity^40^. ND5 mutations have been reported in many mitochondrial diseases such as Leber’s hereditary optic neuropathy (LHON)^41^, mitochondrial encephalopathy^40^, mitochondrial encephalomyopathy with lactic acidosis and strokelike episodes (MELAS)^42^, myoclonic epilepsy with ragged red fibres (MERRF)^43^, and Leigh syndrome^44^. Interestingly, when the ROS level in differentiated PD63 is reduced by growing cells in the presence of the ROS scavenger NAC, a drop in H2AX phosphorylation is observed indicating that the elevation in ROS content has a main role in causing chromosomal DNA damage and DDR activation with possible consequence on genome stability and cellular senescence^45, 46^. Certainly, the observation that increased levels of ROS occur only in PD63 but not in PD67 cells may indicate that a defective mtETC complex is only one of the mechanisms through which mitochondria dysfunction may cause neurodegeneration.

Another aspect emerging from our analysis is the involvement of DROSHA in the assembly of DDR foci in PD63 cells and hence in the cell response to ROS generated by mitochondrial dysfunction. Components of the RNAi machinery and non-coding RNA are emerging as key regulators of several nuclear processes in mammalian cells, including genome surveillance mechanisms^47, 48^. The processing of these DNA damage response RNAs (DDRNAs) is initiated by the endoribonucleases DROSHA and concluded by DICER. Notably, the function of both enzymes, and hence of DDRNAs, is not required for the primary recognition of DNA lesions and the assembly of γH2AX foci. Rather, they act together with γH2AX histone modification spreading for the ensuing recruitment of DDR factors and the assembly of functional DDR foci^13^. In agreement with this, down-regulation of DROSHA hampers the secondary recruitment of p-ATM that is needed for the amplification of DDR signalling. Notably, differently from previous studies in proliferating cells^13, 14^, in terminally differentiated cells DROSHA down-regulation produces a significant reduction in the number of detectable γH2AX foci. This is likely to reflect the fact that differentiated cells do not activate ATR, i.e. the other apical checkpoint kinase involved in the amplification of H2AX phosphorylation^6^ and ATM is the main kinase responsible for DDR activation in neurons *in vivo* ^49^.

So far the involvement of DROSHA has been demonstrated only in the context of DNA damage induced by replication stress in oncogene-induced senescent cells or upon exposure to exogenous sources of DNA damage such as X-ray irradiation or sequence specific DSBs produced by the action of a restriction enzymes^10, 13, 14^. Our data extend the involvement of DROSHA in the DDR activation triggered by increased levels of ROS generated by mitochondrial dysfunction. The observation that DROSHA is needed for the assembly and the persistence of DDR foci in differentiated non-proliferating cells adds another piece of evidence to the physiological role played by this factor in the response to endogenous genotoxic insults in differentiated cells of our body, thus possibly in the prevention of cellular senescence and organismal aging. This might be of major relevance for understanding the molecular mechanism behind PD and probably other neurodegenerative diseases associated with oxidative DNA damage.

It has been recently shown that random DNA damage generated by ionizing radiation, genotoxic drugs, or H_2_O_2_ preferentially persists at telomeres over time^50^ leading to lasting DDR activation. This phenomenon seems to involve a role of TRF2, a telomeric DNA binding protein, in the suppression of DNA repair at telomeres^51^. To this regards it is interesting to notice that telomeric DDRNAs generated by DROSHA, have been recently reported to control DDR activation at de-protected telomeres^52^, which are a major source of cellular senescence in human cells and in aged primates^53^ and may be involved in neurodegenerative disorders. Finally, DROSHA is required for physiological brain development and neuronal function^54^ and loss-of-function mutations of this enzyme have been associated with other neurodegenerative diseases such as frontotemporal lobar degeneration and amyotrophic lateral sclerosis^55^ and Fragile X-associated tremor/ataxia syndrome^56^. Thus, the here reported observation that DROSHA is crucial for the cell response to genomic DNA damage induced by ROS generated by mitochondrial dysfunction opens a new perspective to investigate sporadic neurodegenerative disorders.

## Methods

### Cell lines growth and differentiation

The neuroblastoma SH-SY5Y cell line (ATCC® CRL-2266™) was maintained in monolayer culture in DMEM supplemented with 4mM L-glutamine, 50 μg/ml gentamicin (Sigma) (complete medium, CM) and 10% FBS (Sigma). PD61, PD63 and PD67 cybrid cell lines^22, 24^ were obtained by Patricia A. Trimmer, the Morris K. Udall Parkinson’s Disease Research Center of Excellence and Department of Neurology, University of Virginia, USA, (VCU MTA-13-290) and maintained in monolayer culture in DMEM supplemented with 10% FBS, 4mM L-glutamine, 100 μg/ml pyruvate, 50 μg/ml uridine and 50 μg/ml gentamicin as previously described^21^.

For neuronal differentiation proliferating SH-SY5Y and cybrids (40.000 cells in 60 mm dishes) were grown in Opti-MEM medium (Gibco, Life technologies) with 10% FBS for 24 hours prior to siRNA transfection. Then 200 pmol of siRNA against splicing regulator *PTBP1* were transfected using 20 μl RNAiMAX Reverse Transfections Lipofectamine (Thermo Fisher Scientific) following the manufacturer’s protocol. Transfection was performed using siGENOME ON-TARGETplus SMART pool siRNA (Dharmacon), which consists of four nucleotide sequences targeted to the *PTBP1* gene. A non-targeting pool of four different oligos (Dharmacon) was used as a control. Two cycles of siRNA transfection were performed with 24-hour intervals. Six hours after the second transfection, cells were transferred to CM with 1% FBS, N-2 supplement (Invitrogen) and all-trans retinoic acid (ATRA 3 μg/ml; Sigma Aldrich). After 5 days, cells were grown for further 7 days in serum-free medium containing brain derived neurotrophic factor (BDNF 50 ng/ml; Peprotech), beta-nerve growth factor (NGF 10 ng/ml; Peprotech), Neuregulin 1 beta 2 protein (NRG 10 ng/ml; Abcam), Vitamin D3 (VitD3 9.35 μg/ml; Sigma Aldrich)^57^ and N-2 supplement. The culture medium was changed every 3–4 days. We did not observed significant difference in the survival of cybrids throughout the differentiation protocol.

When requested, N-acetyl-cysteine (NAC) or N-acetyl-alanine (NAA) (Sigma-Aldrich) was freshly added every two days to the differentiation medium at 1 or 5 mM final concentration. A 100 mM stock solution was prepared in water, pH was adjusted to 7.4 with NaOH. ATP content was measured on viable differentiated cells according to the ATP bioluminescent somatic cell assay kit (SIGMA-ALDRICH). Lipid peroxidation was determined by the reaction of MDA with thiobarbituric acid to form a colorimetric (532 nm) product, according to the Lipid peroxidation (MDA) assay kit (SIGMA-ALBRICH).

### Viral Infection

Lentiviruses were produced by transfecting 10 cm dish HEK293 by calcium phosphate method with pMDL g/p RRE (gag-pol elements), pVSVG (envelope elements), pRSV-REV (reverse transcriptase) plasmids and the transfer pLKO vector expressing shDROSHA (DROSHA mRNA targeting sequence 5’-CCAGTGCTAATAACAGCAGTA-3’) or expressing shDICER (DICER mRNA targeting sequence 5’-CCACACATCTTCAAGACTTAA-3’) or pLKO empty vector as control. Viral particles were concentrated incubating each 10 cm dish of transfected HEK293 cells with 5 ml DMEM 10% FBS for 24 hours after transfection. Supernatants containing viral particles were collected, filtered and frozen at −80 °C. 1 ml of medium containing viruses was used to infect differentiated PD63 cells plated in a 35 mm dish for 72 hours before fixation and staining.

### RNA isolation and PCR

Total RNA was extracted using the RNeasy Mini Kit (Qiagen) according to the manufacturer’s protocol. 2 μg of DNAse I-treated total RNA was retro-transcribed using Superscript III First-Strand Synthesis System (Thermo Fisher Scientific). An aliquot (1 μl) of RT reaction was PCR amplified in BioRAD T100 Thermal Cycler as previously described^58^ with the following primer pairs: *PTBP2* (F) 5′-GAGTGGGTATGCCTGGAGTCT-3′, *PTBP2* (R) 5′-GTTTCCATCAGCCATCTGTATTA-3′. Real time quantitative PCR was performed using QuantiTect SYBR Green PCR kit (Qiagen) as previously described^59^ with the following sets of primers: *GAPDH-1* (F) 5′-TCTCCTCTGACTTCAACAGCGACA-3′; *GAPDH-1* (R) 5′-GACAAAGTGGTCGTTGAGGGCAAT-3′; *RPLP0* (F) 5′-TTCATTGTGGGAGCAGAC-3′, *RPLP0* (R) 5′-CAGCAGTTTCTCCAGAGC-3′; *H2AX* (F) 5′-TCAGCTCTCCCTCCATCTTC-3′; *H2AX* (R) 5′-TGTGCCTGTTACCAAGTGCT-3′.

### Western blotting

Cell lysates were prepared with Laemmli buffer supplemented with protease inhibitors (complete tablet; Roche) and phosphatase inhibitors (PhosSTOP tablet; Roche) and analysed by Western blotting with the following primary antibodies: anti-γH2AX (Abcam); anti-PTB (Abcam); anti-nPTB (Abcam); anti-βIII Tubulin (Abcam); anti-Tyrosine Hydroxylase (Abcam); anti-GAPDH (Thermo Scientific); anti-MAP2 (Novusbio); anti-DROSHA (Cell Signaling); anti-DICER (Sigma); anti-H2AX (Millipore). Primary antibodies were revealed with peroxidase-conjugated goat anti-mouse or anti-rabbit antibodies (Jackson Immunoresearch Laboratories) and enhanced chemiluminescence system (Super Signal West Pico Pierce or Super Signal West Dura Extended). βIII Tubulin antibody was revealed with alkaline phosphatase-conjugated goat anti-chicken antibody (Santa Cruz) with a colorimetric assay.

### Immunofluorescence

Cells grown on glass coverslips were fixed in 4% paraformaldehyde as previously described^59^. Primary antibodies were: anti-γH2AX polyclonal antibody (Abcam), anti-53BP1 monoclonal antibody (Bethyl Laboratories), anti-pATM monoclonal antibody (Rockland). The secondary antibodies were: fluorescein isothiocyanate (FITC)-conjugated goat anti-mouse and tetramethyl rhodamine isocyanate (TRITC)-conjugated goat anti-rabbit immunoglobulin G (Jackson Immunoresearch laboratories). Nuclei were stained with 0.1 μg/ml 4′,6-diamidino-2-phenylindole (DAPI, Sigma). ROS were detected with CellROX® Green Reagent (Life Technologies) following manufacturer’s protocol. Images were acquired with a wide-field epifluorescence microscope (Olympus IX71) equipped with PlanApo 60X/1.40NA oil immersion objective. Photomicrographs were taken with digital camera Cool SNAP ES (Photometrics) and data acquisition was done using the MetaMorph software (Universal Imaging Corporation). Images were analyzed by the automated software CellProfiler^60^. Numbers of γH2AX foci per nucleus were quantified by applying an ad hoc designed pipeline that, based on size and fluorescence intensity of γH2AX foci relative to the background signal, recognizes and counts their number in each DAPI positive cell nucleus. ATM activation in γH2AX positive damaged regions was quantified by using a pipeline that creates a mask around the nuclear γH2AX-positive damaged area and identify the same area in the corresponding pictures from the channel used for ATM^pS1981^ (p-ATM) staining. Mean fluorescence intensity for p-ATM staining in γH2AX-positive damaged area of control or DROSHA KD cells was plotted as measured by CellProfiler software. More than 300 nuclei were analysed in each of three independent experiments. Dot-plots show the quantification of one representative experiment.

### Sequencing of mitogenomes

Genomic DNAs from PD61, PD63, and PD67 cybrid cell lines were extracted following standard phenol/chloroform methods. The entire mitogenome of each cybrid was amplified with a set of 11 overlapping PCR fragments and sequenced by standard chain termination sequencing with 33 nested oligonucleotides^29^. Before sequencing, PCR fragments were purified using the ExoSAP-IT® enzymatic system. The obtained mitogenomes were aligned to the revised Cambridge reference sequence (rCRS)^61^, assembled, and compared using Sequencher 5.0 (Gene Codes). The PD63 mitogenome was also assessed by Next Generation Sequencing (NGS) with an Illumina MiSeq^®^. In this case, two overlapping PCR fragments (7,959 bp and 9,244 bp), covering the whole mtDNA sequence were used. Primer pairs for the long range PCR reactions were, 5871(F) 5′-GCTTCACTCAGCCATTTTACCT-3′ and 13829 (R) 5′-AGTCCTAGGAAAGTGACAGCGA-3′; 13477 (F) 5′-GCAGGAATACCTTTCCTCACAG-3′ and 6151(R) 5′-ACTAGTCAGTTGCCAAAGCCTC-3′. The amplification was performed with 10–50 ng of template DNA in 50 µl of reaction mix containing 1X GoTaq® LongPCR Master Mix (Promega) and 0.2 µM of each primer, according to manufacturer’s instructions. The PCR program included an initial denaturation step at 94° for 2 minutes, 30 cycles with the following thermal profile: 94 °C for 30 seconds, 55 °C for 30 seconds, 65 °C for 9 minutes and a final extension step at 72 °C for 10 minutes. The two PCR products were purified with Wizard® SV Gel and PCR Clean-Up System (Promega) according to manufacture’s instructions and quantified with a Quantus™ Fluorometer (Promega). A total amount of 1.5 ng of PCR product (0.75 ng for each PCR) was used for the set up of a sequencing library with the Nextera® XT DNA sample preparation kit (Illumina) following the manufacturer’s protocol. Sequencing reactions were carried out on a MiSeq (Illumina) by using the MiSeq Reagent Nano Kit, v2 (300 cycles). On-board software created results in FASTQ format, which were analysed with the Geneious software (version 8.1). This software was used to compare the mitogenome sequence with the rCRS and to create a report of sequence variants (nucleotide substitutions and indels). The average depth of the obtained reads was about 8000X.

For the conservation analysis of amino acid positions, the ND5 amino acid sequences from wide range of eukaryotes were retrieved from uniprot (http://www.uniprot.org) and aligned with the EBI tool MUSCLE (http://www.ebi.ac.uk/tools/msa/muscle/).

### Data availability

Data set generated during the current study are available in the GenBank repository under the accession numbers KY498629 (PD61); KY498630 (PD63); KY498631 (PD67).

## Electronic supplementary material

Supplementary Information
