# Supplementary material for: A missense MT-ND5 mutation in differentiated Parkinson Disease cytoplasmic hybrid induces ROS-dependent DNA Damage Response amplified by DROSHA
Source: Sci Rep. 2017 Aug 25;7:9528. doi: 10.1038/s41598-017-09910-x (PMC5573376; doi:10.1038/s41598-017-09910-x)
Supplement: Supplementary file 1 — Supplementary Information [file 41598_2017_9910_MOESM1_ESM.pdf]

A missense MT-ND5 mutation in differentiated Parkinson Disease cytoplasmic hybrid induces ROS-dependent DNA Damage Response amplified by DROSHA.

Daniela Pignataro[1], Sofia Francia[1], Francesca Zanetta[1], Giulia Brenna[1], Stefania Brandini[2], Anna Olivieri[2], Antonio Torroni[2], Giuseppe Biamonti[1], Alessandra Montecucco[1]*

[1]Istituto di Genetica Molecolare, Consiglio Nazionale delle Ricerche (CNR) Pavia 27100, Italy
[2]Dipartimento di Biologia e Biotecnologie "L. Spallanzani", Università di Pavia, Pavia 27100, Italy

*Correspondence to montecucco@ogm.cnr.it

Sample	nmoles / 200μl extract
SH-SY5Y	0.799
PD67	1.034
PD63	2.076

Supplementary Table 1S: Lipid peroxidation was determined by the reaction of malondialdehyde (MDA) with thiobarbituric acid to form a colorimetric (532 nm) product, proportional to MDA present. MDA nmoles were calculated with the Lipid Peroxidation (MDA) assay kit (Sigma-Aldrich) according to the manufacturer procedures.

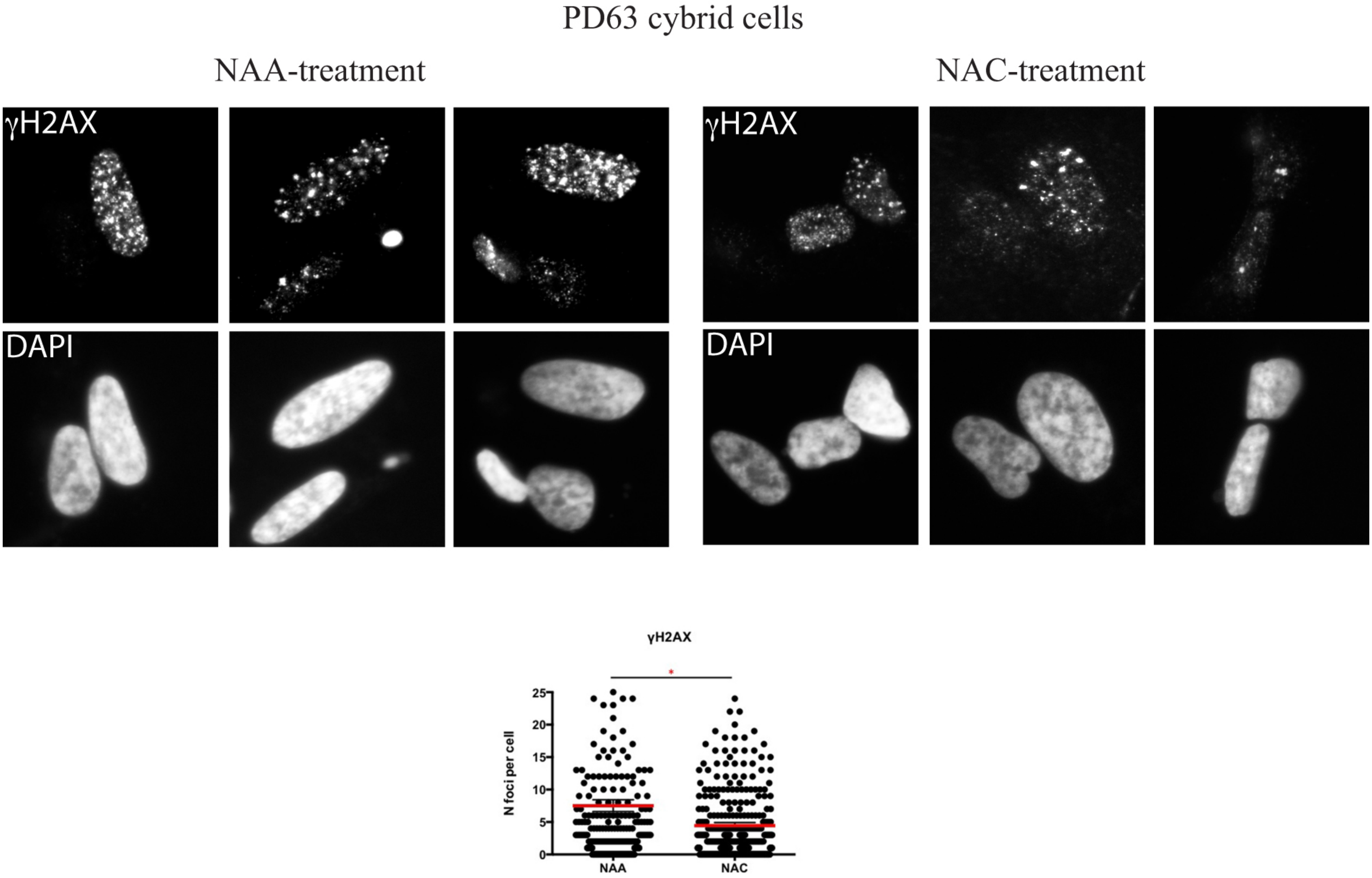

Supplementary Fig. 1S: The number of γH2AX foci per nucleus in NAA and NAC treated PD63 cells was quantified with CellProfiler as described in Material and Methods. Red lines indicate the means of γH2AX foci per nucleus. For these analyses more than 250 cells for each condition were analysed; *= $p< 0.05$.

A: Western blotting **B**: PCR

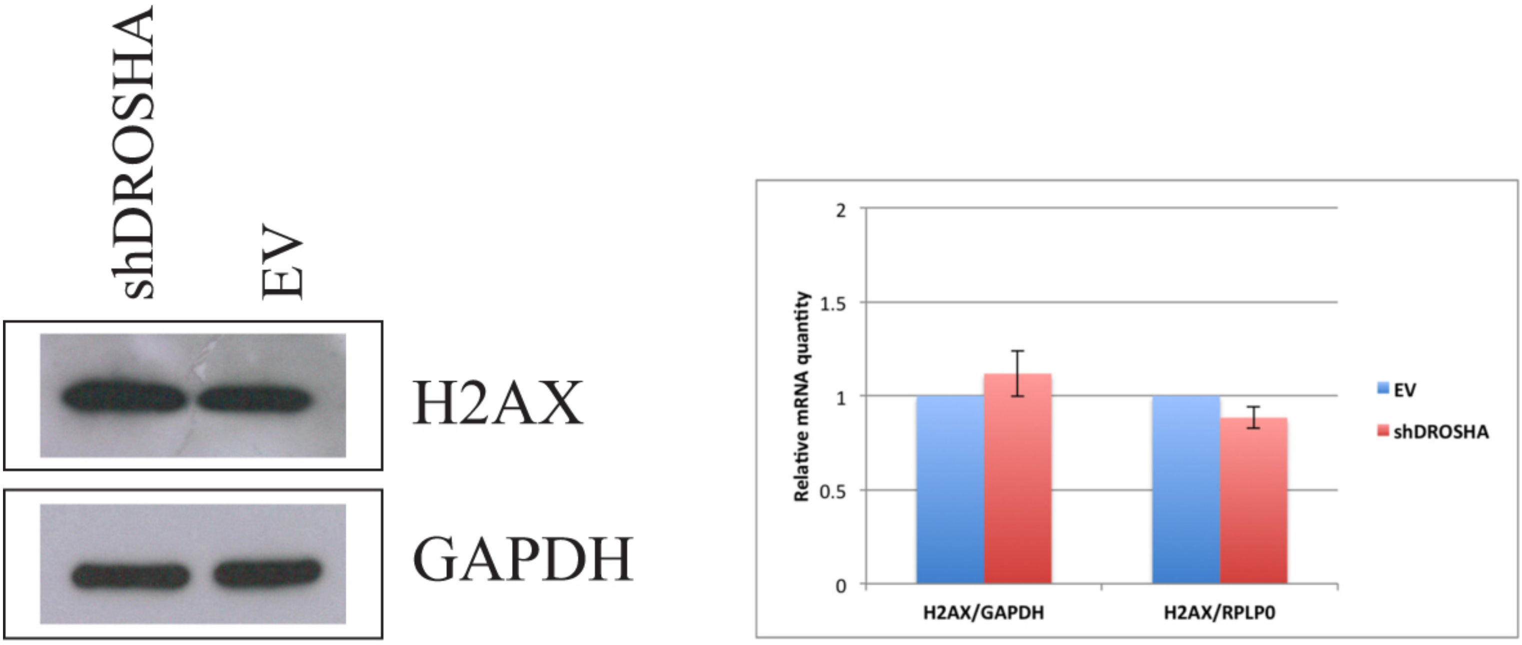

Supplementary Fig. 2S: (A) H2AX protein level in PD63 infected with lentiviruses expressing shDROSHA or the empty vector (EV); GAPDH, loading control. (B) The relative amount of H2AX transcript in the same cells was normalized to the level of GAPDH and RPLPO mRNA.

PD63 cybrids

A: Western Blotting

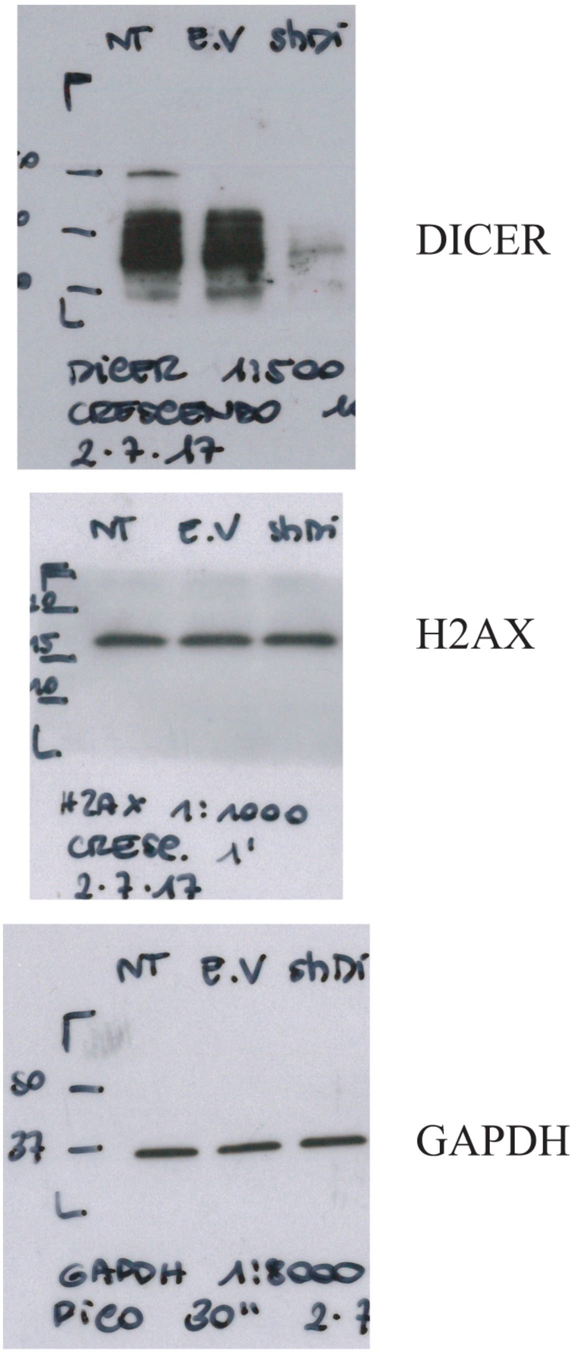

B: Analysis of DDR foci

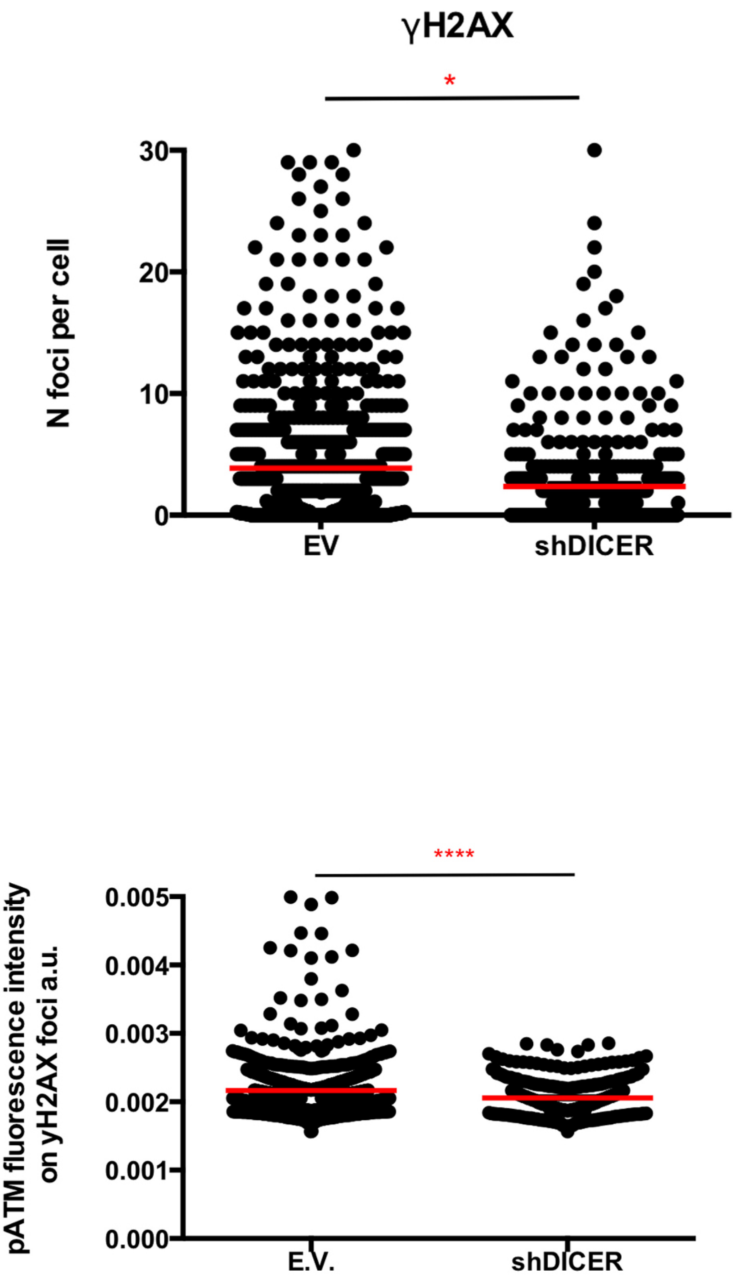

Supplementary Fig. 3S: Effect of DICER knock-down on DDR foci in PD63 cells. (A) Western blot analysis of DICER and H2AX levels in PD63 cells untreated (NT), infected with lentivirus expressing the empty vector (EV) or shDICER. GAPDH, loading control. (B) The number of γH2AX foci per nucleus in PD63 infected with lentiviruses expressing the empty vector (EV) or shDICER was quantified with CellProfiler as described in Methods. Red lines indicate the means of γH2AX foci per nucleus. More than 500 cells for each condition were analysed *=p<0.03. Quantification of the ATMpS1981 (pATM) in γH2AX foci in the same cells. Red lines indicate the mean of pATM focus intensity in the γH2AX-positive damaged area. More than 500 cells for each condition were analysed **** = p< 0.0001.

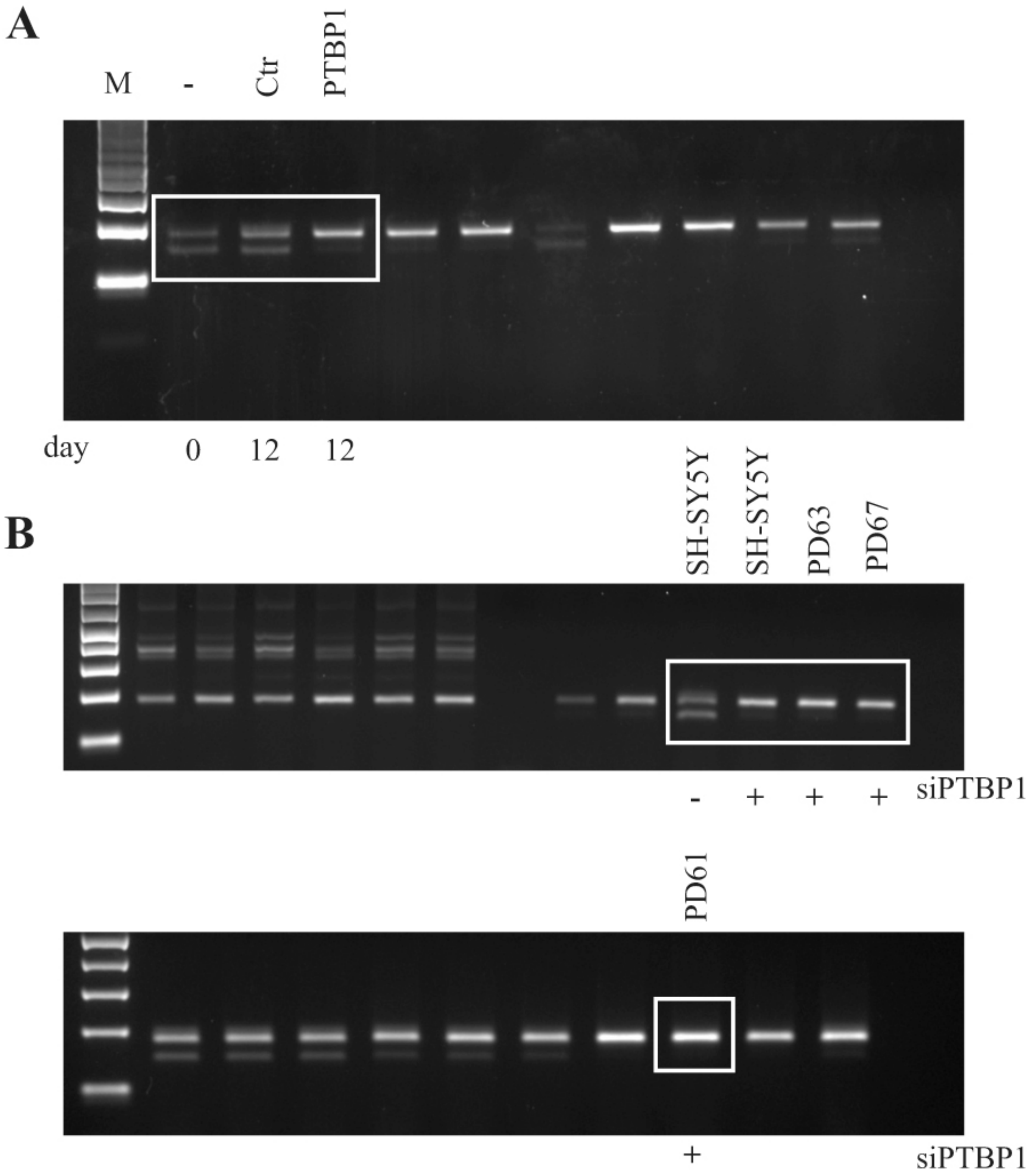

Figure 4S: Original agarose gels of the alternative splicing profiles of *PTBP2* exon 10 as described in Fig.1 Panels A and B. White rectangles indicate the lanes assembled in the Fig.1.

A

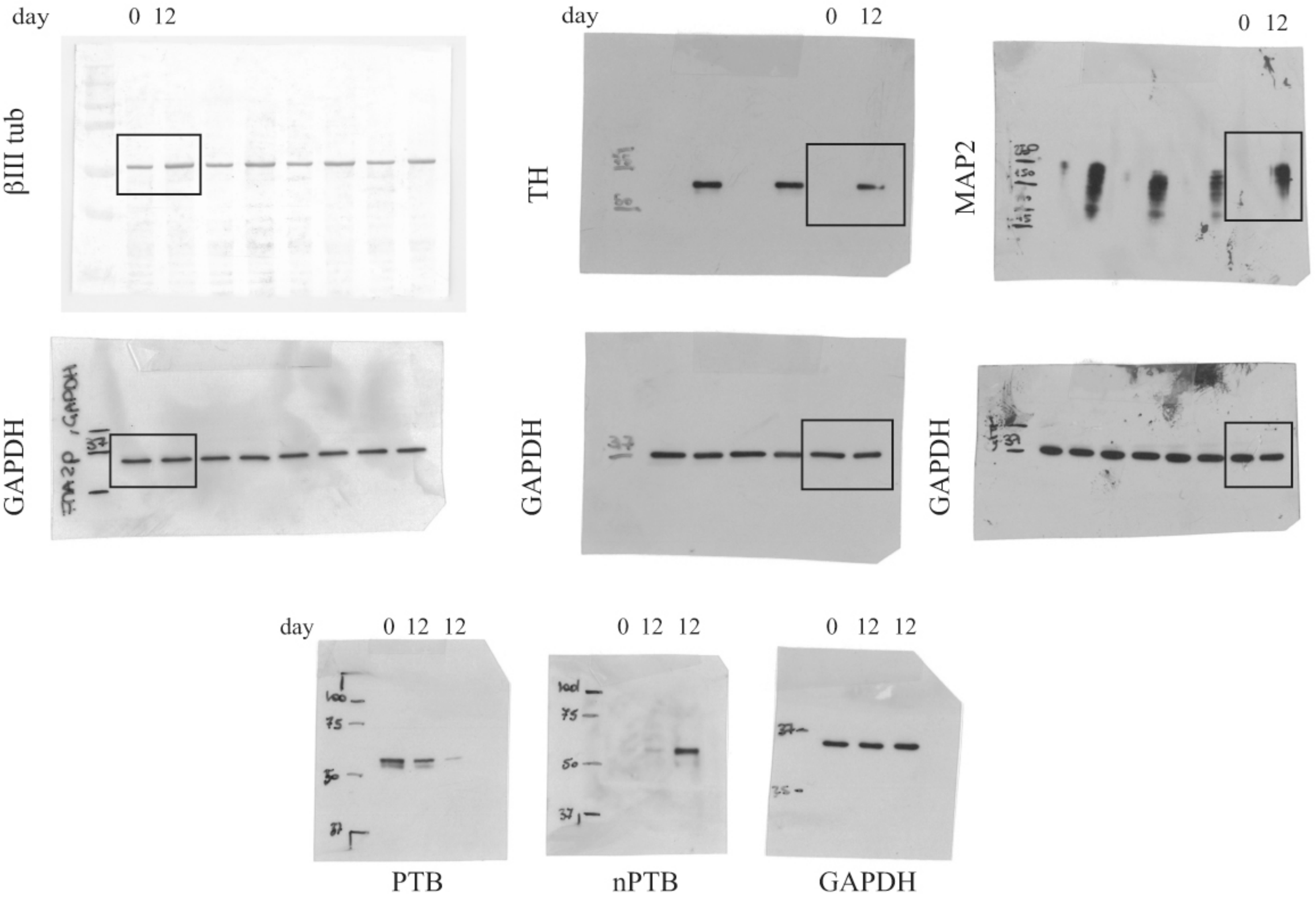

B

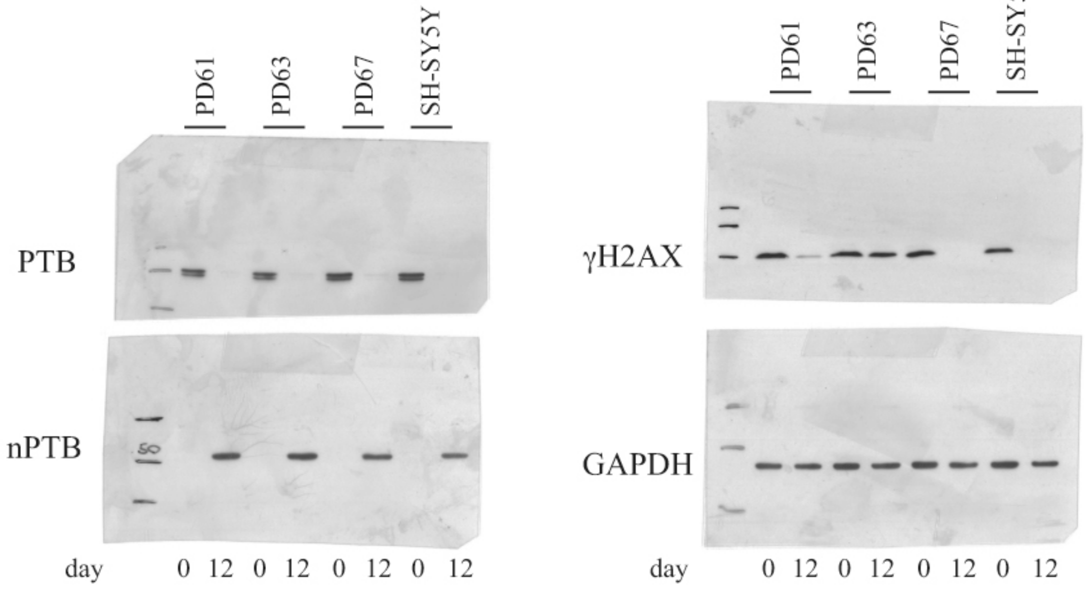

Supplementary Fig 5S: Full-length blots of Fig. 1. Black rectangles indicate the lanes assembled in Fig.1.

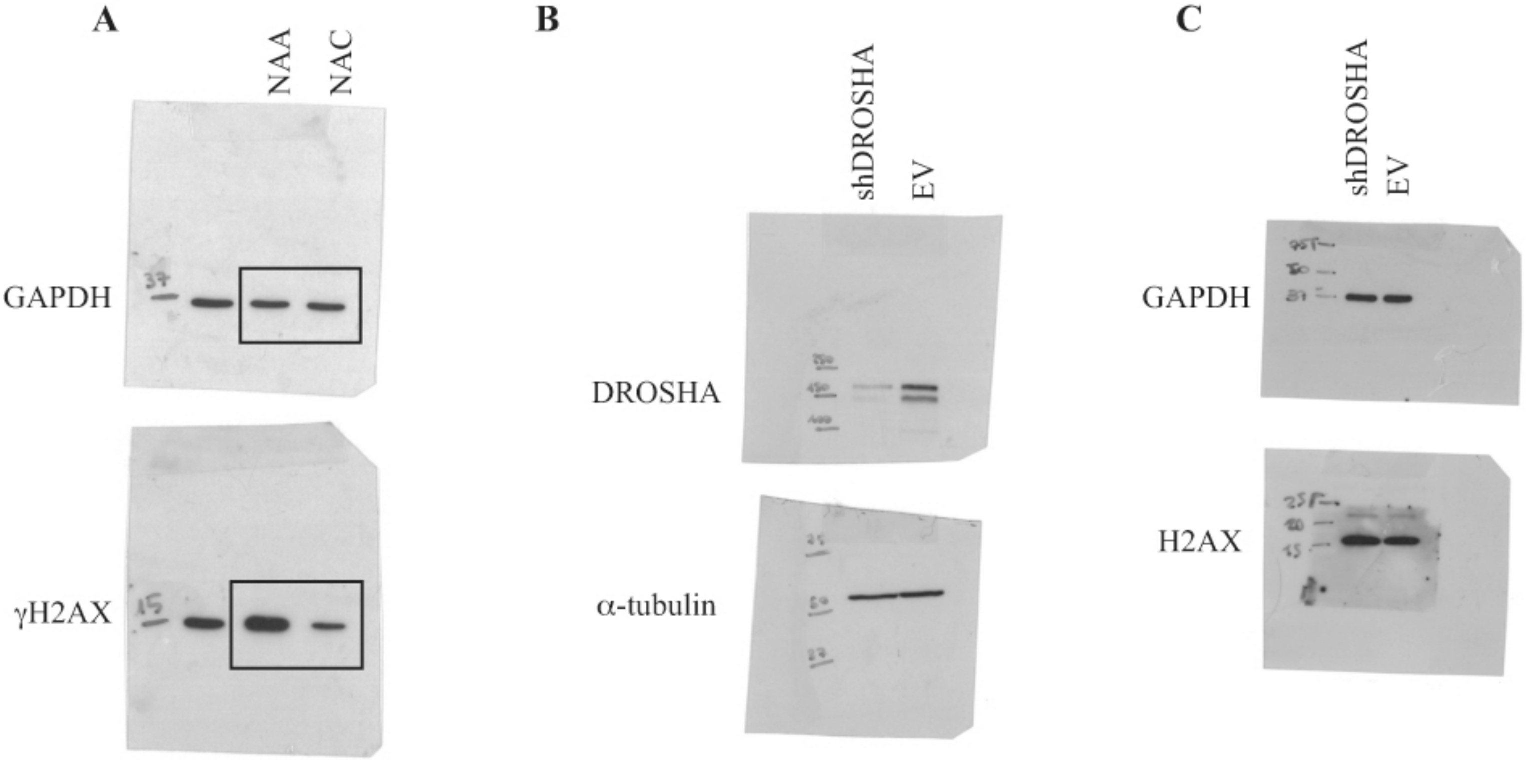

Supplementary Fig. 6S: Full lenght blots: (**A**) Fig. 4, black rectangles indicate the lanes assembled in the figure. The membrane was cut at 25 kD MW marker and the two part were incubated with the indicated antibodies (**B**) Fig.5, extracts were run on a precast 4-15% gel (BioRad) and the membrane was cut between the markers 75 and 100 kD. The two part of the membrane were incubated with the indicated antibodies. (**C**) Supplementary Fig. 2S, the membrane was cut at 25 kD and the two part were incubated with the indicated antibodies.
